# Human‐specific insights into candidate genes and boosted discoveries of novel loci illuminate roles of neuroglia in reading disorders

**DOI:** 10.1111/gbb.12899

**Published:** 2024-05-16

**Authors:** Wen‐Hua Wei, Shaowei Ma, Bo Fu, Ranran Song, Hui Guo

**Affiliations:** ^1^ Centre for Biostatistics, Division of Population Health, Health Services Research and Primary Care The University of Manchester Manchester UK; ^2^ Hebei Key Laboratory of Children's Cognition and Digital Education and School of Foreign Languages Langfang Normal University Langfang China; ^3^ School of Data Science Fudan University Shanghai China; ^4^ Department of Maternal and Child Health and MOE (Ministry of Education) Key Laboratory of Environment and Health, School of Public Health, Tongji Medical College Huazhong University of Science and Technology Wuhan China

**Keywords:** dyslexia, human‐specific, neuroglia, neuronal plasticity, phenotyping, reading disorders

## Abstract

Reading disorders (RD) are human‐specific neuropsychological conditions associated with decoding printed words and/or reading comprehension. So far only a handful of candidate genes segregated in families and 42 loci from genome‐wide association study (GWAS) have been identified that jointly provided little clues of pathophysiology. Leveraging human‐specific genomic information, we critically assessed the RD candidates for the first time and found substantial human‐specific features within. The GWAS candidates (i.e., population signals) were distinct from the familial counterparts and were more likely pleiotropic in neuropsychiatric traits and to harbor human‐specific regulatory elements (HSREs). Candidate genes associated with human cortical morphology indeed showed human‐specific expression in adult brain cortices, particularly in neuroglia likely regulated by HSREs. Expression levels of candidate genes across human brain developmental stages showed a clear pattern of uplifted expression in early brain development crucial to RD development. Following the new insights and loci pleiotropic in cognitive traits, we identified four novel genes from the GWAS sub‐significant associations (i.e., *FOXO3*, *MAPT*, *KMT2E* and *HTT*) and the Semaphorin gene family with functional priors (i.e., *SEMA3A*, *SEMA3E* and *SEMA5B*). These novel genes were related to neuronal plasticity and disorders, mostly conserved the pattern of uplifted expression in early brain development and had evident expression in cortical neuroglial cells. Our findings jointly illuminated the association of RD with neuroglia regulation—an emerging hotspot in studying neurodevelopmental disorders, and highlighted the need of improving RD phenotyping to avoid jeopardizing future genetic studies of RD.

## Graphical abstract

Genetic candidates for dyslexia were critically assessed using human‐specific genomic features and functional information relevant to human brain development. Most candidates carried human‐specific features, but only a small proportion had evidence of regulating human cortical morphology, uplifted expression in early brain and human‐specific expression in cortical neuroglia, jointly highlighting neuronal plasticity.

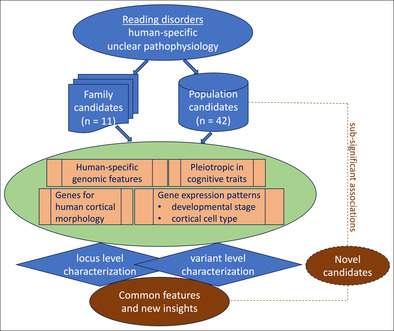

## 1 INTRODUCTION

Reading disorders (RD) are human‐specific neuropsychological conditions associated with decoding printed words and/or reading comprehension.^1^, ^2^ RD were initially referred to as Dyslexia (MIM 600202)—a medical term first coined by German ophthalmologist Rudolf Berlin in 1887, where “dys” stands for difficulty and “lexia” for words, meaning to be difficult or slow in reading and spelling words.^3^, ^4^ RD are highly inheritable but can be modified by environmental factors and their complex interactions with inherited genetic variants during early development.^5^, ^6^, ^7^ Since reading difficulties become identifiable at school in general,^8^, ^9^ RD are related to age of onset, with an estimated prevalence between 5% and 12% of school‐aged children worldwide.^6^ However, genetic studies of RD so far have identified only a handful of candidate genes segregated in families and 42 loci from genome‐wide association study (GWAS) that jointly provided little clues of the pathophysiology of RD.^1^, ^7^, ^10^

Indeed, there are many processes that had happened before RD becoming evident but were unaccounted for in the genetic association studies, particularly those in early cognition and language development in infancy^11^, ^12^ and early childhood.^9^ Such processes each may involve complex gene–environment interactions and jointly form the so called development plasticity.^12^ For example, children carrying inherited genetic variants of RD can develop ways of managing reading difficulties and subsequently achieve reading performance around the average or above, whereas those without RD genetic variants could become poor performers in reading tests because of bad habits and/or psychological barriers in reading developed during the early stage of life. Besides, heterogeneity of definitions of RD across disciplines could have jeopardized genetic studies of RD where the dichotomous case–control settings were often used,^3^, ^4^ including self‐reported RD cases and controls.^10^, ^13^, ^14^

One way to explore values of the available genetic discoveries is to understand their functional relevance to brain development that is considered be to the key to RD. Functional studies of some familial variants identified from family studies have provided some key insights into genetic regulation of RD.^1^, ^15^ However, such familial variants tend to be rare and thus are difficult to replicate in populations.^1^, ^5^ On the other hand, advances in uncovering genomic changes evolutionarily specific to humans have resulted in a rich source of human‐specific genomic information^16^, ^17^, ^18^ that can also be used to derive novel insights from the candidate variants into diseases of interest.^2^, ^19^, ^20^ One well‐known example is the *FOXP2* gene that was first discovered as a candidate responsible for a severe language and speech disorder in a family study.^21^ *FOXP2* was further found to be important in human language development and has regions containing human‐specific elements but without recent positive selection among diverse human populations.^22^ Further studies indicated that *FOXP2* had human‐ as well as primate‐specific expression in human brain^23^ that was upregulated by human‐specific regulatory changes in only two neuronal subtypes.^18^ Another example is the RD candidate gene *DCDC2* carrying a highly polymorphic transcription regulatory element READ1 with human‐specific attributes.^24^, ^25^ It is therefore desired to derive additional human‐specific insights from *DCDC2* and other RD candidate genes/variants.

Furthermore, it has been well received that common human brain disorders probably share some genetic basis^26^, ^27^ as partially illuminated by evolutionary genomics theories such as diametric diseases.^2^ Exploring the shared basis for human‐specific regulatory insights, particularly pleiotropic loci associated with multiple neuropsychiatric traits,^28^ could be useful to recover variants for RD from previous sub‐significant associations given relatively low power in the studies owing to small samples and/or RD definition issues. We, therefore, conducted this study to explore any human‐specific genomic features in candidate genes/variants identified previously (Data S1 to S3) and derive new insights into any human‐specific regulatory mechanisms. We used data published in our previous study on primate‐specific genomic information^20^ and recent studies of human brain evolution and development^18^, ^23^, ^29^ as the sources of human‐specific information. We quote genomic positions in GRCh38/hg38 throughout.

## 2 MATERIALS AND METHODS

### 2.1 Rationale

Given the human‐specific nature of RD and the neuropsychological characteristics associated with early brain development, a natural step to address the challenge in understanding the underlying pathophysiology from the limited genetic findings is to borrow information from related disciplines. If the genetic findings are real, they will probably have human‐specific regulation like *FOXP2* and will be involved in brain development with functions relevant to RD. Therefore, we characterized the RD genetic findings using existing information on human‐specific genomic features, genes associated with human cortical morphology, genes pleiotropic in cognitive traits, gene expression patterns across cortical cell types as well as brain developmental stages, to identify common features and new insights into the pathophysiology of RD. The identified features and insights were applied to a highly relevant gene family and sub‐significant associations of the original GWASs to uncover novel loci hidden owing to the low power of detection.

Characterization of the RD familial and population loci/variants was conducted at the locus level and then the variant level as appropriate, which was described in detail below followed by a subsection of novel loci discovery. For convenience, all genomic coordinates were quoted in the GRCh38/hg38 version and conversions of coordinates in the GRCh37/hg19 version were made beforehand using the LiftOver function in the Genome Browser (https://genome.ucsc.edu/cgi-bin/hgLiftOver).

### 2.2 Input data

#### 2.2.1 RD genetic findings

RD candidate genes and variants identified from family studies were collated mainly from the comprehensive review by Graham and Fisher^1^ in addition to a few studies published after the review^15^, ^30^, ^31^ (Data S1). A total of 42 genome‐wide significant associations were collected as population candidate genes and variants identified from the GWAS of RD^10^ (Data S2).

#### 2.2.2 Existing genetic genomic information

A total of 431 loci pleiotropic in neuropsychiatric traits were collated from a latest study of pleiotropy^28^ (Data S3).

For human‐specific genomic information, primate‐specific information regions (PSIregion) were adopted from our previous study^20^ (Data S4), newly discovered human‐specific elements from a study of human brain evolution,^18^ including human‐specific differentially expressed gene (hsGene) per cell type, human‐specific differentially accessible cis‐regulatory element (hsCRE) per cell type, hsCREs within the 500 kilobase vicinity of an hsGene (hsCRE‐hsGene), human accelerated regions (HARs), cis‐regulatory elements (CREs) overlap with HARs (CRE‐HAR), modern human‐specific variants (MHS), CREs overlap with MHS (CRE‐MHS) (Data S5 to S11, respectively).

#### 2.2.3 Cortical morphology and brain related functional information

- Protein‐coding genes significantly associated with cortical area or thickness (corticalGene) were quoted from a study of human cortical morphology^29^ (Data S12).
- Gene expression in dorsolateral prefrontal cortex of adult brains of four primate species: humans, chimpanzees, rhesus macaques and marmosets, using the existing single‐nucleus transcriptome data generated to study evolution of the primate dorsolateral prefrontal cortex.^23^ Data were extracted and visualized using an interactive online tool *Cross‐species dot plots* http://resources.sestanlab.org/PFC/.
- Gene expression across human brain developmental stages was examined using the data from the Atlas of the Development of Human Brain (http://brainspan.org/rnaseq/search/index.html). Data were extracted from the Atlas for each candidate gene of interest and visualized in Microsoft Excel to generate plots.

#### 2.2.4 GWAS summary statistics to identify novel loci

The GWAS summary statistics of the top 10,000 associated SNPs of the Doust's study^10^ were downloaded freely from https://doi.org/10.7488/ds/3465. The full summary statistics of the GWAS of quantitatively assessed reading‐ and language‐related skills in up to 34,000 people^32^ were downloaded freely from https://www.genlang.org/downloads.html.

### 2.3 Characterization at the locus level

This subsection concentrated on two questions: (1) would the RD candidates and the neuropsychiatric pleiotropic loci colocalize with human‐specific genomic features and/or be functionally relevant to brain development? (2) would RD familial candidates differ from the GWAS candidates as expected?

For each RD candidate locus or variant (Data S1 and S2), tests of colocalization with human‐specific genomic features or pleiotropic loci were performed one by one by looking for overlaps with the latter. A colocalization was recorded if there was an overlap between the genomic location of the RD candidate under test and the genomic location of a target feature or pleiotropic locus. This was performed by checking their chromosome and start and end genomic positions using a self‐developed script (available on request). Tests of functional relevance were also performed for each RD candidate locus or variant using the gene assigned to search against each functional dataset (Data S5 and S12). The test results were stored in Tables S1 and S2 accordingly. For comparison purposes, pleiotropic loci were also tested for colocalization with human‐specific genomic features and functional relevance and the results were stored in Table S3.

Subsequently, summary statistics were derived from the test results to address the two questions above, where for each RD candidate locus or variant, one colocalization with either of the human‐specific features tested would mark the association of the locus or variant with human‐specific feature. Association of each of the pleiotropic loci with human‐specific features was marked in the same way. Differences between familial and GWAS candidates were examined using Chi‐square tests. Common features and novel insights were derived during the comparisons.

### 2.4 Characterization at the variant level

RD variants associated with human‐specific features and modern human‐specific variants (i.e., MHS) colocalized with RD candidate loci were characterized in this subsection, to evaluate likelihood of causality of these variants in the context of evolutionary genetics (i.e., age of the mutant allele and allele frequencies in African and European populations), human‐specific regulatory information, expression in cortical cell type and deleterious effects. The MHS variants emerged in modern human populations normally beginning with the African populations,^18^ and hence were used to derive evolutionary and functional insights into the RD candidate genes. For example, the allele frequency would increase in European populations if an MHS variant was under positive selection against the ancestral African populations or decrease if under negative selection.

### 2.5 Discovery of novel loci

Common features and functional insights derived from the characterization processes above were applied to two sets of data to discover novel loci for RD:

- The Semaphorin gene family with *SEMA3C* and *SEMA3F* identified as RD candidates and with functional priors highly relevant to human brain developmental issues.^23^, ^33^ The locus level characterization was performed for the remaining members of the gene family and those which shared the common features were considered as novel loci of RD.
- Sub‐significant associations (*p* < 1.0 × 10^−6^) in previous GWASs of RD^10^ and reading skills.^32^ These were first filtered by overlapping with the loci pleiotropic in cognitive traits and the remaining were characterized at the locus level, and those which shared the common features were considered as novel loci of RD.

### 2.6 Additional genomic analysis tools

- Since the vast majority of RD candidate variants were identified from samples with European ancestry, NCBI dbSNP (Build 155, https://www.ncbi.nlm.nih.gov/projects/SNP/snp_summary.cgi) was used to extract variant allele frequencies for European, African and the aggregated populations to show allele frequency changes owing to selection.
- To characterize RD candidate variants, we further extracted their Combined Annotation‐Dependent Depletion (CADD) score – a widely used measure of variant deleteriousness^34^ from https://cadd.gs.washington.edu/ and variant evolutionary age^35^ from https://human.genome.dating/.

## 3 RESULTS

Nearly all the listed 11 RD familial candidate loci and the 42 GWAS loci colocalized with certain human‐specific elements (Table 1, Tables S1 and S2, Data S4 to S12). The only exception is the familial candidate gene *DYX1C1* colocalized with only a primate‐specific transcription unit. Of the 431 neuropsychiatric pleiotropic loci, 89.3% also colocalized with certain human‐specific elements (Table 1, Table S3), suggesting human‐specific elements are prevalent in brain‐related disorders. Interestingly, 45.2% (i.e., 19) of the 42 GWAS loci were among the 780 unique loci responsible for human cortical morphology development (Table S2, Data S12),^29^ whereas the proportion was ~28% in both the familial and pleiotropic loci (Table 1).

**TABLE 1:** Associations of RD candidate loci with human‐specific features and cortical morphology.^a^

	Familial (11)	GWAS (42)	Pleiotropic (431)
Human‐specific feature	90.9%	100%	89.3%
Brain morphology	27.3%	45.2%	27.8%

^a^ Numbers of loci in brackets; *DYX1C1* was the only familial candidate with no overlaps with human‐specific features but one primate‐specific transcription unit.

**FIGURE 1:**
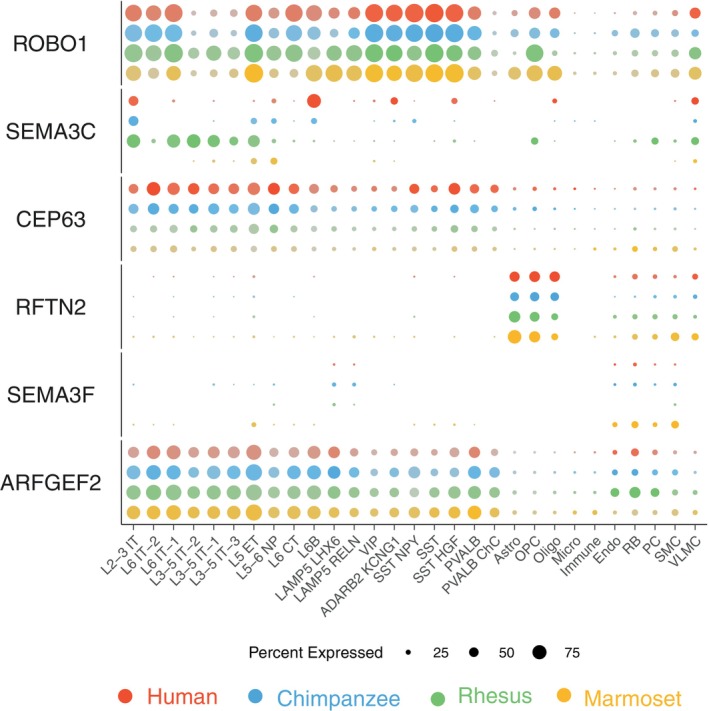
Gene expression of cortical morphology associated familial and GWAS candidates across 29 subclasses of cells from dorsolateral prefrontal cortex of adult humans, chimpanzees, rhesus macaques, and marmosets. Data extracted using *Cross‐species dot plots* (http://resources.sestanlab.org/PFC/) with a minimum expression at the 5% level; the 29 subclasses grouped in 4 major classes: glutamatergic excitatory neuron (10, L2‐3 IT to L6B), GABAergic inhibitory neuron (9, LAMP5 LHX6 to PVALB ChC), glial cell (4, Astro to Micro) and non‐neural cell (6, Immune to VLMC); Astro, astrocyte; ChC, chandelier cells; CT, corticothalamic; Endo, endothelial cells; ET, extratelencephalic; IT, intratelencephalic; L, layer; Micro, microglia; NP, near‐projecting; Oligo, oligodendrocytes; OPC, oligodendrocyte precursor cells; PC, pericyte; RB, red blood lineage cells; SMC, smooth muscle cells; VLMC, vascular leptomeningeal cells.

The associations of three of the 19 GWAS loci associated with cortical morphology were reported^10^ to have replicated in at least two independent cohorts: *RFTN2*, *SEMA3F* and *ARFGEF2* (Tables S2 and S4). Their expression levels across cell subclasses from prefrontal cortices of four primates (i.e. adult humans, chimpanzees, rhesus macaques, and common marmosets),^23^ in contrast to that of three cortical morphology associated familial loci *ROBO1*, *SEMA3C* and *CEP63* (Table S1) were shown in Figure 1. These genes all expressed with certain but different human‐specific patterns in various cortical cell types, suggesting complex mechanisms in regulating human prefrontal cortices. *SEMA3C* had clearly human‐specific expression in a number of cell types including increased expression in human oligodendrocytes like *RFTN2*, whereas *CEP63* was the only one with slightly increased expression in human microglia like *FOXP2* ^23^ (Figures 1 and S2a).

The familial candidates colocalized with zero pleiotropic neuropsychiatric loci, one HAR and three MHS, in contrast 19 pleiotropic loci, 10 HARs and 21 MHS colocalized by the GWAS candidates. Although the only difference in colocalization with pleiotropy was significant (Pearson's Chi‐squared test, X‐squared = 4, 65, *df* = 1, *p* = 0.03), our observations reinforced the notion that familial signals tend to be family‐specific whereas GWAS loci tend to capture population signals that are likely pleiotropic and regulatory (e.g., HAR).^20^, ^36^ Of the 19 pleiotropic loci colocalized with RD GWAS candidates, 8 were replicated in cognitive traits only (Table S2).

Table 2 characterized familial variants with human‐specific features tagged and MHS variants residing within either familial or GWAS candidate loci with replication. The familial variants rs144517871 and rs143835534, intronic of *SEMA3C*, were a lot more recent than most MHS variants (except for rs540764086), but appeared more frequently in European populations than those previously reported,^15^ possibly because of strong positive selection occurred in less than two thousand generations (or 50,000 years). In contrast, all the MHS variants underwent negative selection in European populations. The variants in familial loci were generally more frequent than those in GWAS loci in African populations. Furthermore, most hsCREs tagged by the familial loci upregulated gene expression in the Oligodendrocyte cell type, while those by the GWAS loci tended to downregulate gene expression in the Oligodendrocyte and additional cell types. Nonetheless, neither of the listed variants alone could be causal of RD owing to relatively low CADD scores (Table 2).

**TABLE 2:** RD familial variants directly tagged by human‐specific features and modern human‐specific variants residing within either familial or GWAS candidate loci with replication.

Variant	chr	Position	Allele	Gene	European	African	Adjacent human‐specific element	Age	CADD
rs76737144	7	80908760	A > G	*SEMA3C*	0.0046	0.1394	chr7:80907172‐80907800, up, Oligodendrocyte	37,898	0.0
rs144517871^a^	7	80918075	A > C	*SEMA3C*	0.0137	0.0024	chr7:80918336‐80920558, up, Oligodendrocyte	1338	16.9
rs143835534^a^	7	80918259	T > C	*SEMA3C*	0.0134	0.0024	chr7:80918336‐80920558, up, Oligodendrocyte	1805	2.0
rs73434215	7	111775190	T > C	*DOCK4*	0.0009	0.1903	chr7:111794184‐111795181, down, L3‐5_RORB_1	23,661	0.6
rs60412264^b^	21	46447615	A > G	*DIP2A*	0.0765	0.1985	chr21:46442761‐46443421, up, Oligodendrocyte	44,209	3.6
rs59308388^b^	21	46447658	C > T	*DIP2A*	0.0764	0.2024	chr21:46442761‐46443421, up, Oligodendrocyte	47,345	0.7
rs113032385	2	197838082	C > T	*PLCL1*	0.0002	0.0458	chr2:197836182‐197836472 (human‐specific insertion)	49,349	5.1
rs113638606	4	151746036	T > C	*GATB*	0.0003	0.0426	chr4:151762915‐151763509, down, Oligodendrocyte	33,783	2.2
rs113801043	4	151753240	A > C	*GATB*	0.0003	0.0431	chr4:151762915‐151763509, down, Oligodendrocyte	34,173	0.3
rs112761935^b^	6	16940454	A > G	*STMND1*	0.0000	0.0784	chr6:16964157‐16965977, down, Astrocyte	45,548	0.3
rs6910825^b^	6	16993475	C > T	*STMND1*	0.0011	0.0807	chr6:16964157‐16965977, down, Astrocyte	39,367	0.5
rs540764086^b^	12	120619105	G > C	*CABP1*	0.0000	0.0044	in chr12:120617778‐120620271, down, Astrocyte	836	1.8
rs77336065^b^	14	99276804	G > T	*BCL11B*	0.0000	0.0044	in chr14:99276678‐99278056, down, L4‐5_RORB_1	10,089	1.7
rs74474830	17	36592859	T > C	*DHRS11*	0.0351	0.1592	in chr17:36590929‐36593324, down, Astrocyte	20,261	1.4

*Note*: two *SEMA3C* sequence variants marked with superscript a; other variants listed were all modern human‐specific either within familial or GWAS loci, where GWAS loci were either replicated in at least two cohorts or overlapped with pleiotropic loci replicated in cognitive traits; variants were intronic except for those marked with superscript b in which cases adjacent genes were listed; chr, chromosome; European (African), minor allele frequency in European (African) population cited from dbSNP; age, median age in generations (25 years per generation) calculated using combined data and Joint clock; adjacent human‐specific element, all hsCRE except for one noted, all within 28‐kilobases to the corresponding variants, either upregulate or downregulate gene expression in a cell type separated by comma; CADD, Combined Annotation‐Dependent Depletion—a widely used measure of variant deleteriousness.

**FIGURE 2:**
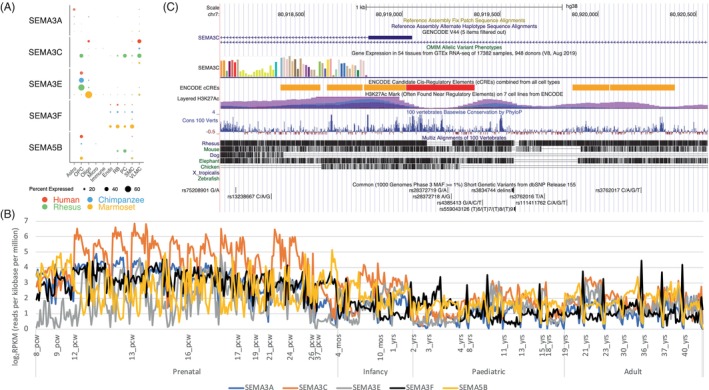
Gene expression of candidates of Semaphorin gene family in glial cell types (A) and across different stages of human brain development (B) and the human‐specific cis‐regulatory element tagged by two *SEMA3C* sequence variants (C). Astro, astrocyte; Endo, endothelial cells; Micro, microglia; mos: months; Oligo, oligodendrocytes; OPC, oligodendrocyte precursor cells; PC, pericyte; pcw, post‐conception weeks; RB, red blood lineage cells; SMC, smooth muscle cells; VLMC, vascular leptomeningeal cells; yrs, years; (A): expression data extracted using Cross‐species dot plots (http://resources.sestanlab.org/PFC/) with a minimum expression at the 5% level; (B): expression data extracted from the Atlas of the Development of Human Brain (http://brainspan.org/rnaseq/search/index.html); (C) plot generated by Genome Browser (https://genome.ucsc.edu/cgi‐bin/hgGateway).

Considering both *SEMA3C* and *SEMA3F* (Figure 1) belong to Semaphorin family, we further examined expression of both the genes and additional members of the gene family *SEMA3A*, *SEMA3E* and *SEMA5B* implicated elsewhere^23^, ^33^ (Figure 2). Interestingly, *SEMA3C* and *SEMA3A* expressed in oligodendrocyte and astrocyte respectively, *SEMA3E* and *SEMA5G* expressed in oligodendrocyte precursor cells, but none in microglial cells (Figure 2A). *SEMA3C* appeared to be mostly expressed at the prenatal and then infancy stages and reduced expression substantially afterward (Figure 2B). The remaining Semaphorin family genes expressed with a similar pattern but at a reduced level across the prenatal and infancy stages (Figure 2B). The hsCRE chr7:80918336–80920558 tagged by the *SEMA3C* variants rs144517871 and rs143835534 (Table 2), enclosed the exon 1 of the gene, had widely varied levels of conservation (the “Layered H3K27Ac” panel) and overlapped with multiple CREs in different cell types (the “ENCODE cCREs” panel) with clear regulatory evidence (Figure 2C). We further looked into common and rare variants within the hsCRE to explore if rs144517871 and rs143835534 could instead tag rare variants above. We found a few that were evolutionarily younger with lower frequencies in European populations than the two variants above, including rs564263256 that was a mutation in European populations about 261 generations ago and rs73709368 that seemed to be a common variant in African populations about 726 generations ago but underwent strong negative selection in European populations (Table S5).

In contrast, of the three well‐known familial RD candidates (Figure S1), *DCDC2* had little expression in glia cells, while *KIAA0319* expressed in astrocyte and *FOXP2* in microgalia as previously reported^23^ (Figure S1a). *DCDC2* had low expression across human brain developmental stages, whereas *KIAA0319* had relatively high expression throughout. *FOXP2*, like *SEMA3C*, mostly expressed at the prenatal stages then remained lowly expressed (Figure S1b). Most of the *DCDC2* microdeletion was lowly conserved, with only small chunks to be human‐specific (e.g., chr6: 24326600–24326820) (Figure S1c), where the reported candidate rs1417740^37^ appeared to be too common to carry RD risk, and no obvious alternative variants could be nominated (Table S6).

**FIGURE 3:**
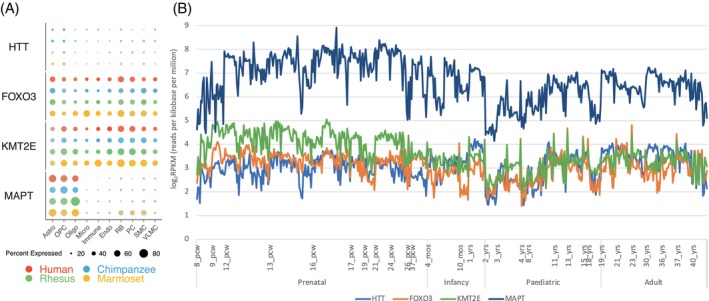
Gene expression of four identified novel loci in glial cell types (A) and across different stages of human brain development (B). Astro, astrocyte; Endo, endothelial cells; Micro, microglia; mos, months; Oligo, oligodendrocytes; OPC, oligodendrocyte precursor cells; PC, pericyte; pcw, post‐conception weeks; RB, red blood lineage cells; SMC, smooth muscle cells; VLMC, vascular leptomeningeal cells; yrs, years; (A): expression data extracted using Cross‐species dot plots (http://resources.sestanlab.org/PFC/) with a minimum expression at the 5% level; (B): expression data extracted from the Atlas of the Development of Human Brain (http://brainspan.org/rnaseq/search/index.html).

Considering loci pleiotropic in cognitive traits (Data S3), 9 overlapped with the sub‐significant associations available from two published GWASs^10^, ^32^ (Table 3). Eight of the 9 loci carried certain human‐specific features. Intriguingly, *HTT*, *FOXO3*, *KMT2E* and *MAPT* carried at least one feature regulating gene expression in glial cells. In addition, both *FOXO3* and *MAPT* were involved in cortical area as well as thickness development (Table 3). Furthermore, these genes expressed in glia cells including microglia where *FOXO3* had the highest and *MAPT* the lowest expression level among the four (Figure 3A). These four genes were therefore considered to be novel loci, each with evident expression across the prenatal stages as well as other stages in life, where the expression of *MAPT* was the highest throughout (Figure 3B).

**TABLE 3:** Novel loci identified from sub‐significant GWAS signals by overlapping pleiotropic loci replicated in cognitive traits.^a^

chr	Start	End	Gene	PSIregion	hsCRE	HAR	MHS	corticalGene
1	96195036	96502355	*LINC01787*	hsInsert	No	No	No	No
4	3140934	3240118	*HTT*	hsInsert	Yes, not glia	No	Yes, glia	No
6	19056096	19098182	*intergenic*	No	No	Yes, not glia	No	No
6	97862415	98102413	*MIR2113*	No	No	No	Yes, not glia	No
6	108540061	108606639	*FOXO3*	hsInsert	No	Yes, glia	No	Area, thickness
7	104953266	105085688	*KMT2E*	hsInsert	Yes, glia	No	Yes, glia	No
7	105205928	105421332	*SRPK2*	hsInsert	Yes, not glia	No	No	No
14	98140957	98174050	*LINC02295*	No	No	No	No	No
17	45962818	46018358	*MAPT*	No	Yes, glia	No	No	Area, thickness

^a^ chr, chromosome; start (end), start (end) position of a locus; intergenic, locus in an intergenic region; PSIregion, pre‐defined primate‐specific information region (Data S4); hsCRE, human‐specific cis‐regulatory element (Data S6); HAR, human accelerated region (Data S8); MHS, modern human‐specific variants (Data S10); corticalGene, overlap with gene related to human cortical morphology development (Data S12); hsInsert, human‐specific insertion; No, no overlap; Yes, (not) glia, overlapped human‐specific element (does not) regulate gene expression in a glial cell type.

## 4 DISCUSSION

Leveraging accumulated human‐specific genomic information, we critically assessed candidate genes/variants for reading disorders for the first time. We showed that the vast majority of these candidate loci carried certain human‐specific features, as fresh evidence supporting that human traits likely have human‐specific origins.^2^, ^20^ We further showed that unlike those identified from family studies, the GWAS candidates were population signals likely to harbor human‐specific regulatory elements such as HARs and MHS and carry pleiotropy in neuropsychiatric traits. Concentrating on candidate genes important to human cortical morphology, we observed human‐specific patterns in their expression across adult brain cortical (in particular, neuroglial) cell types. Most of the hsCREs tagged by MHS residing in the candidate loci also had differential accessibility in neuroglial cell types. In addition, expression of the candidate genes across human brain developmental stages highlighted distinct patterns in early brain development crucial to RD as previously predicted.^11^, ^12^ These together rendered new approaches to the assessments and discoveries of novel loci for RD.

Taking the familial candidate *SEMA3C* and GWAS candidate *SEMA3F* as an example, concerning gene expression in adult brain cells alone (Figure 1) could miss their roles in early brain development where expression appeared to be three to four magnitudes (in the log_2_ scale) higher at the prenatal and infant stages than that in adulthood (Figure 2B). Given that *SEMA3C* was involved in the development of the human cortical area and *SEMA3F* in both area and thickness,^29^ it is plausible that carriers of causal variants (yet to identify) would have reduced cortical area and/or thickness, possibly by disrupting *SEMA3C* (e.g., in oligodendrocytes, Figure 2A) and *SEMA3F* expression at the early brain developmental stages, which would have gone missing if considering adult brain only.^23^ It also seems plausible that for genetic causal factors of RD, those with uplifted expression in early brain development (i.e., prenatal and infancy stages) could carry relatively higher risk, in which case *SEMA3C* would have a higher effect than *SEMA3F* and thus segregated only in families but not populations. Of the remaining four genes presented in Figure 1, we also observed high expression of *ROBO1* in the prenatal and infancy stages (Figure S2). Nevertheless, it remains challenging to identify the *SEMA3C* causal variants,^15^ where the causal factors like hsCRE and the increased expression of *SEMA3C* in oligodendrocytes could be useful clues (Figure 2C, Table S5).

To boost discoveries of novel loci, we first examined additional Semaphorin family genes *SEMA3A*, *SEMA3E* and *SEMA5B* together with the known candidates *SEMA3C* and *SEMA3F*, given that Semaphorins were important in regulating neuronal plasticity and diseases^33^ and the former three were identified with primate specific expression patterns previously.^23^ Clearly, *SEMA3A*, *SEMA3E* and *SEMA5B* expressed in a similar pattern as *SEMA3C* but with a lower level of expression during early brain development, suggesting that they could also contribute to risk of RD. For example, *SEMA3A* is a known candidate for Autism and depression^33^ and is also among the 780 unique loci responsible for both cortical area and thickness development.^29^ Nonetheless, why *SEMA3F* was detected in the GWAS but not *SEMA3A*, *SEMA3E* and *SEMA5B* remains an open question.

Utilizing the shared genetic basis for cognitive traits we further identified nine novel loci from the GWAS sub‐significant associations (Table 3, Figure 3). Interestingly, four of the five protein‐coding genes each had at least one human‐specific element regulating gene expression in glial cells (Table 3) and indeed all expressed in glial cell types (Figure 3A), and across early stages of human brain development (Figure 3B). These genes also expressed well in adult brain stages which might explain to some extent their pleiotropy and associations with neurodegenerative disorders such as Huntington's and Parkinson's diseases.^38^, ^39^, ^40^ For example, *MAPT* is a well‐known candidate for Parkinson's disease^41^ and is highly expressed at the early stages of human brain development (Figure 3B). Recent evidence showed neurodevelopmental consequences triggered by structural changes of *MAPT* and subsequently cortical morphology.^42^, ^43^ Similarly, neurodevelopmental evidence was also reported for *KMT2E*,^44^ *HTT* ^45^ and *FOXO3*.^46^ Nevertheless, all the novel loci need replication and further investigation in future studies of RD.

Our results collectively illuminated possible roles of neuroglia in RD risk. Since *FOXP2* ^23^ and *SEMA3C* showed clearly human‐specific expression in microglia and oligodendrocyte, respectively (Figures 2 and S1), we further examined other RD candidates and the novel loci and found replication of the phenomenon in most of those discussed, suggesting possible association of RD with regulation of the candidate gene expression in neuroglial cells. On the other hand, rapid advances in neuroscience have been expanding our understanding of fundamental roles of neuroglia in human brain diseases ranging from neurodevelopmental disorders such as autism to neurodegenerative problems like Parkinson's disease^47^, ^48^, ^49^, ^50^ These roles cover the entire process of human brain, from early development (e.g., neurogenesis and neuronal migration) through to maturation and function maintenance, well illustrating the early concept of neuroglia as a substance that holds nervous parts together and gives the whole its form.^49^ Surprisingly, although widely considered as common and complex neuropsychological conditions,^5^, ^8^ RD was not yet among the reported neuropsychiatric disorders related to aberrant functions of neuroglia.^47^, ^48^, ^49^, ^50^

At least two factors could possibly explain the obvious missing above. First, RD neuroglia focused studies probably barely chose RD cases given the relatively low severity but relatively high complexity imposed by psychological/environmental factors.^3^, ^5^, ^6^, ^9^ Second, at the point of sampling RD cases it could have been too late to capture sufficient neuroglial signals because RD‐associated neural problems might have happened in pre‐ and peri‐natal stages^12^, ^51^, ^52^ long before RD diagnosis. For the same reasons, we argue that the neuronal migration hypothesis of RD, previously criticized for lack of robust evidence from either rodent models or human studies,^7^ should not be dismissed but instead deserve further investigation that may benefit from the most updated methods to characterize roles of neuroglia in the early development of RD. Our argument is further supported by previous and our current findings that (a) rodent models are useful but not sufficient for human specific problems^53^ so cannot be used to dismiss the hypothesis regardless; (b) human microglia has confirmed roles in regulating neuronal migration^51^, ^52^, ^54^ and neuronal migration is considered as a risk factor for autism^55^; (c) RD is associated with Semaphorins family genes (Figure 2) and with gene expression in oligodendrocytes and/or oligodendrocyte precursor cells (Table 2, Figures 1, 2, 3), while the class 3 Semaphorins (i.e., *SEMA3A*, *SEMA3C*, *SEMA3F*) could inhibit the migration of oligodendrocyte precursor cells from the ventricular zones of the brain to the central nervous system^33^ and differentiation into mature oligodendrocytes that are essential to form the myelin sheath for efficient transmission of the nervous impulse.^49^

Our study highlights a key challenge to future studies of RD: how to improve phenotyping given dynamic nurture and complex interactions with genetic defects that probably happen at early stages of human brain developments. Such genetic defects are unlikely severe, probably beneath a disease liability threshold^5^ or otherwise might have been diagnosed as other neurodevelopmental disorders. New methods have become available to address issues related to age of onset of risk development.^56^, ^57^ However, further efforts are required to tackle the key challenge, particularly when environmental and social factors become major determinants of disease risk.^2^ A further investigation of weaker sub‐significant associations, that is, those with *p*‐values weaker than the arbitrary cutoff of 1.0 × 10^−6^ used in this study, could identify additional novel candidates that, however, need to be validated in independent studies as well. In conclusion, using human‐specific genomic information and genetic regulatory basis shared across neuropsychiatric disorders, we critically assessed genetic candidates for RD and derived important new insights into pathophysiology of RD and identified novel candidate loci that jointly illuminated possible roles of neuroglia in early RD risk development.

## CONFLICT OF INTEREST STATEMENT

The authors declare no competing interests.

## Supporting information


**Figure S1:** Gene expression of RD candidates *DCDC2*, *KIAA0319* and *FOXP2* in glial cell types (a) and across different stages of human brain development (b) and genomic view of the *DCDC2* microdeletion (chr6:24325133–24,327,581) of harboring the polymorphic transcription regulatory element READ1 (c). Astro, astrocyte; Micro, microglia; Oligo, oligodendrocytes; OPC, oligodendrocyte precursor cells; Endo, endothelial cells; RB, red blood lineage cells; PC, pericyte; SMC, smooth muscle cells; VLMC, vascular leptomeningeal cells; pcw: post‐conception weeks; mos: months; yrs: years; (a): expression data extracted using Cross‐species dot plots (http://resources.sestanlab.org/PFC/) with a minimum expression at the 5% level; (b): expression data extracted from the Atlas of the Development of Human Brain (http://brainspan.org/rnaseq/search/index.html); (c) plot generated by Genome Browser (https://genome.ucsc.edu/cgi-bin/hgGateway).

**Figure S2:** Gene expression of RD familial candidates *ROBO1* and *CEP63*, GWAS candidates *RFTN2* and *ARFGEF2* in glial cell types (a) and across different stages of human brain development (b). Astro, astrocyte; Micro, microglia; Oligo, oligodendrocytes; OPC, oligodendrocyte precursor cells; Endo, endothelial cells; RB, red blood lineage cells; PC, pericyte; SMC, smooth muscle cells; VLMC, vascular leptomeningeal cells; pcw: post‐conception weeks; mos: months; yrs: years; (a): expression data extracted using Cross‐species dot plots (http://resources.sestanlab.org/PFC/) with a minimum expression at the 5% level; (b): expression data extracted from the Atlas of the Development of Human Brain (http://brainspan.org/rnaseq/search/index.html).

**Data S1:** Candidate genes and/or variants identified from family studies.
**Data S2:** Genome‐wide significant associations identified from the 23andMe GWAS and suggestive variants from previous GWAS.
**Data S3:** List of 431 pleiotropic loci associated with neuropsychiatric traits.
**Data S4:** Non‐overlap PSI regions converted with hg38 coordinations.
**Data S5:** Human‐specific differentially expressed genes (hsGene) per cell type.
**Data S6:** Human‐specific cis‐regulatory element (hsCRE) with differential accessibility per cell type.
**Data S7:** Human‐specific differentially accessible cis‐regulatory element (hsCRE) within the 500 kilobase vicinity of an human‐specific differentially expressed gene (hsGene).
**Data S8:** Publicly available human accelerated regions (HARs) and newly identified cortical HARs.
**Data S9:** Cis‐regulatory elements (CREs) overlap with human accelerated regions (HARs).
**Data S10:** Modern human‐specific variants (MHS) evolved after the split from Neanderthals and Denisovans.
**Data S11:** Cis‐regulatory elements (CREs) overlap with modern human‐specific variants (MHS).
**Data S12:** 780 unique genetic loci associated with human cortical morphology.

**Table S1:** Annotation of familial variants and loci with human‐specific features.
**Table S2:** Annotation of GWAS variants and loci with human‐specific features.
**Table S3:** Annotation of pleiotropic loci with human‐specific features.
**Table S4:** Replication of GWAS loci associated with cortical morphology.
**Table S5:** Variants within the *SEMA3C* hsCRE chr7:80918336‐80920558.
**Table S6:** Variants within the *DCDC2* microdeletion.
