# Supplementary figures and images for: Human‐specific insights into candidate genes and boosted discoveries of novel loci illuminate roles of neuroglia in reading disorders
Source: Genes Brain Behav. 2024 May 16;23(3):e12899. doi: 10.1111/gbb.12899 (PMC11097622; doi:10.1111/gbb.12899)

a

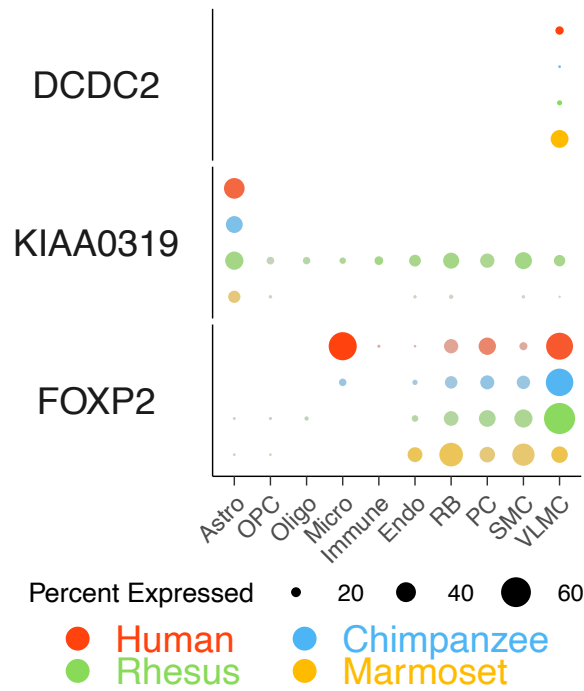

c

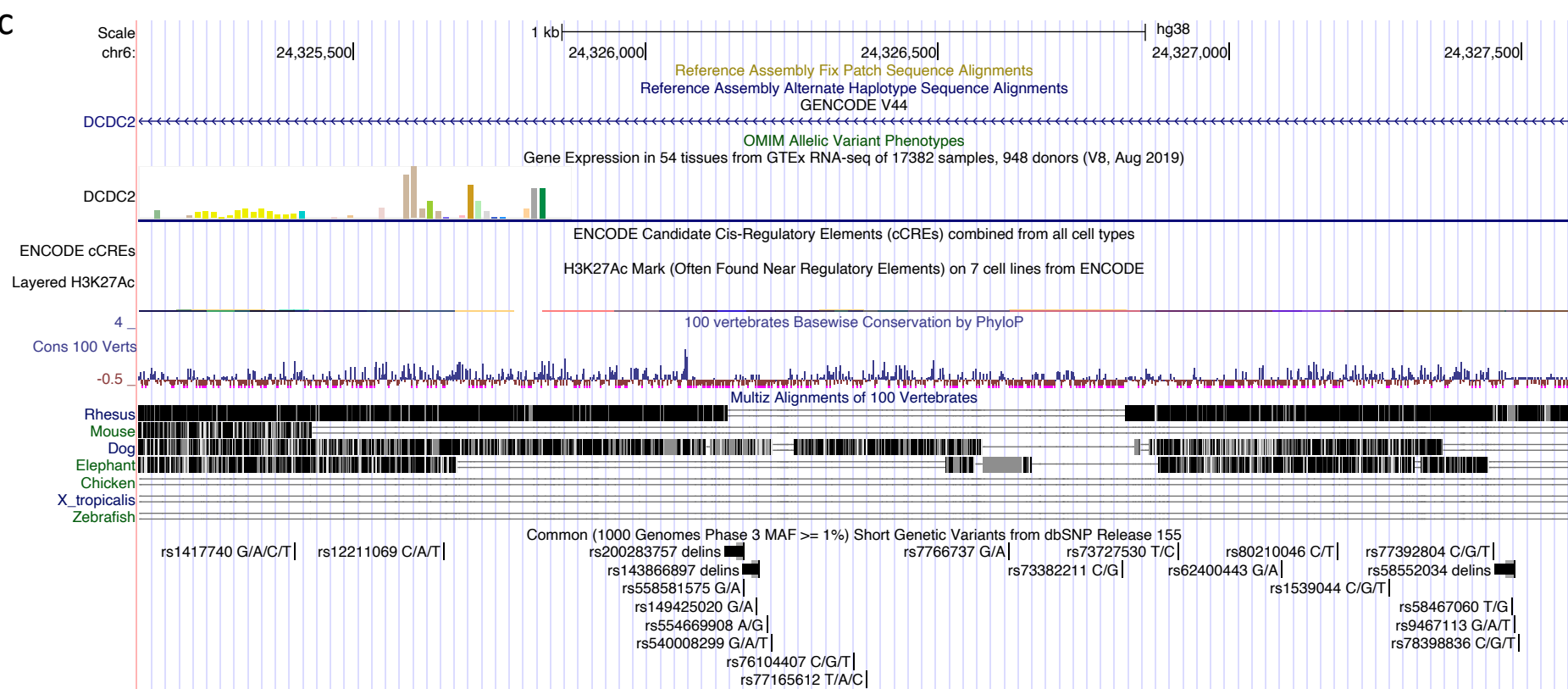

b

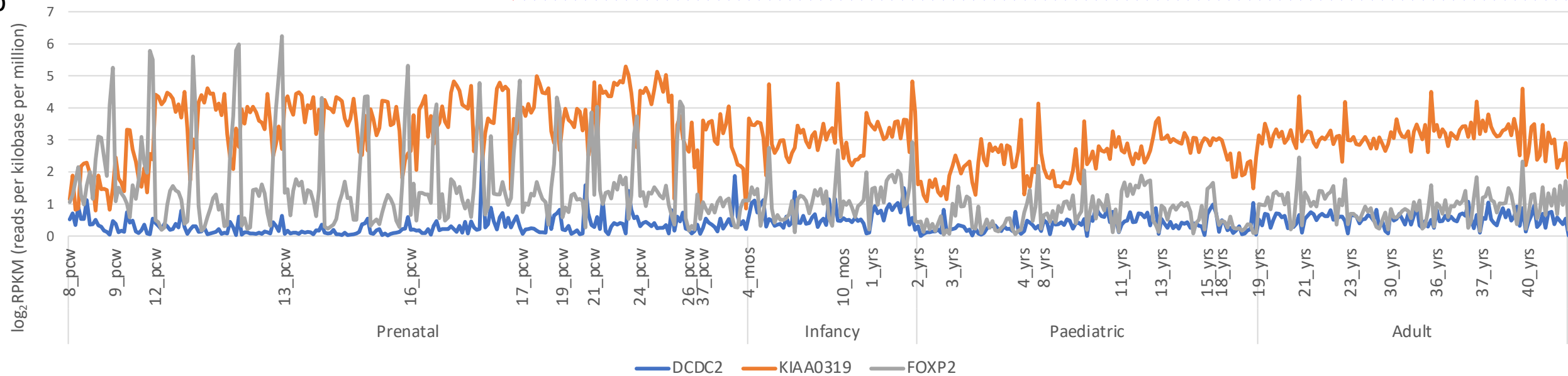

Supplement: Supplementary file 1 — Figure S1: Gene expression of RD candidates DCDC2, KIAA0319 and FOXP2 in glial cell types (a) and across different stages of human brain development (b) and genomic view of the DCDC2 microdeletion (chr6:24325133–24,327,581) of harboring the polymorphic transcription regulatory element READ1 (c). Astro, astrocyte; Micro, microglia; Oligo, oligodendrocytes; OPC, oligodendrocyte precursor cells; Endo, endothelial cells; RB, red blood lineage cells; PC, pericyte; SMC, smooth muscle cells; VLMC, vascular leptomeningeal cells; pcw: post‐conception weeks; mos: months; yrs: years; (a): expression data extracted using Cross‐species dot plots (http://resources.sestanlab.org/PFC/) with a minimum expression at the 5% level; (b): expression data extracted from the Atlas of the Development of Human Brain (http://brainspan.org/rnaseq/search/index.html); (c) plot generated by Genome Browser (https://genome.ucsc.edu/cgi-bin/hgGateway). [file GBB-23-e12899-s001.pdf]

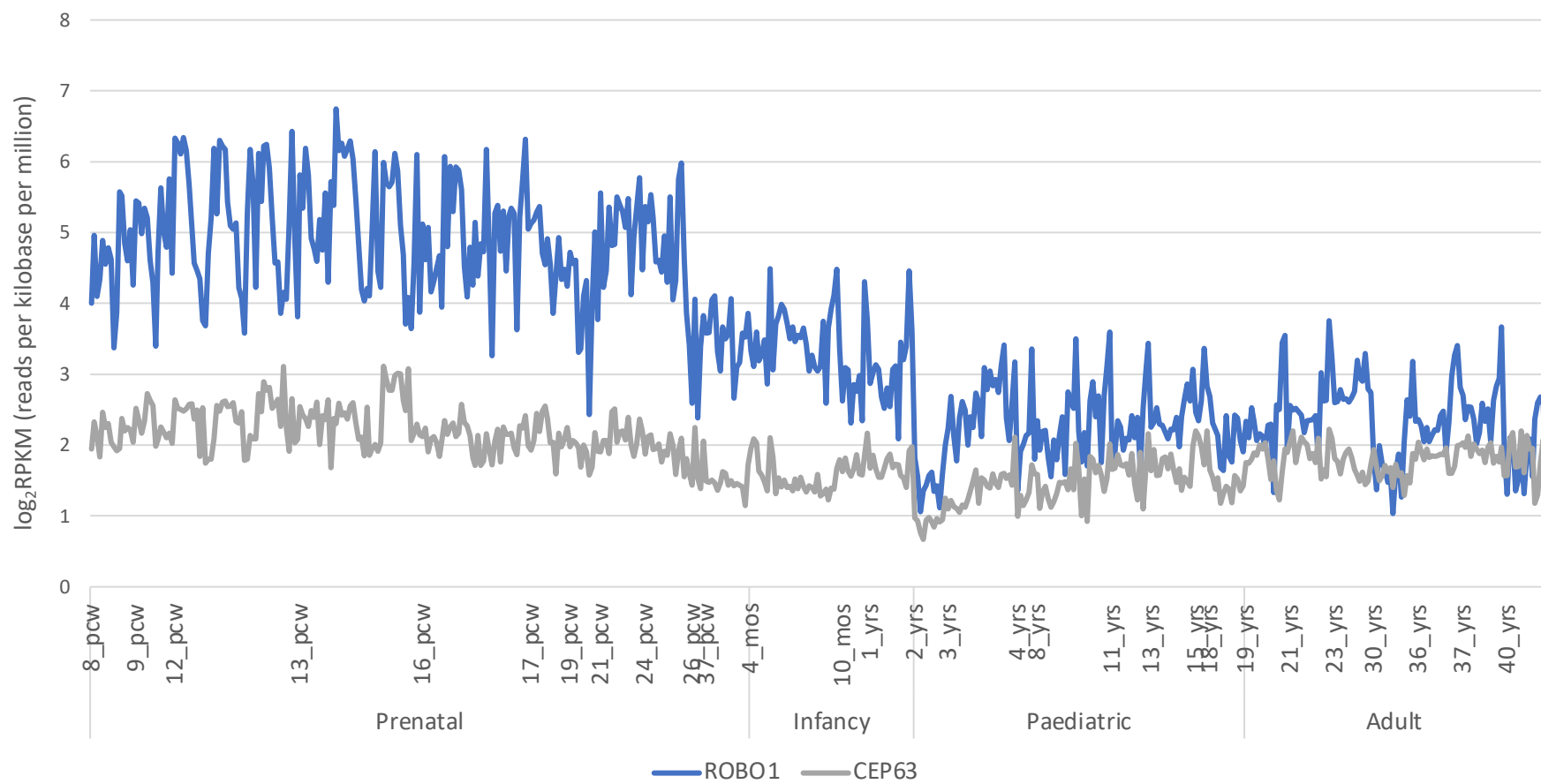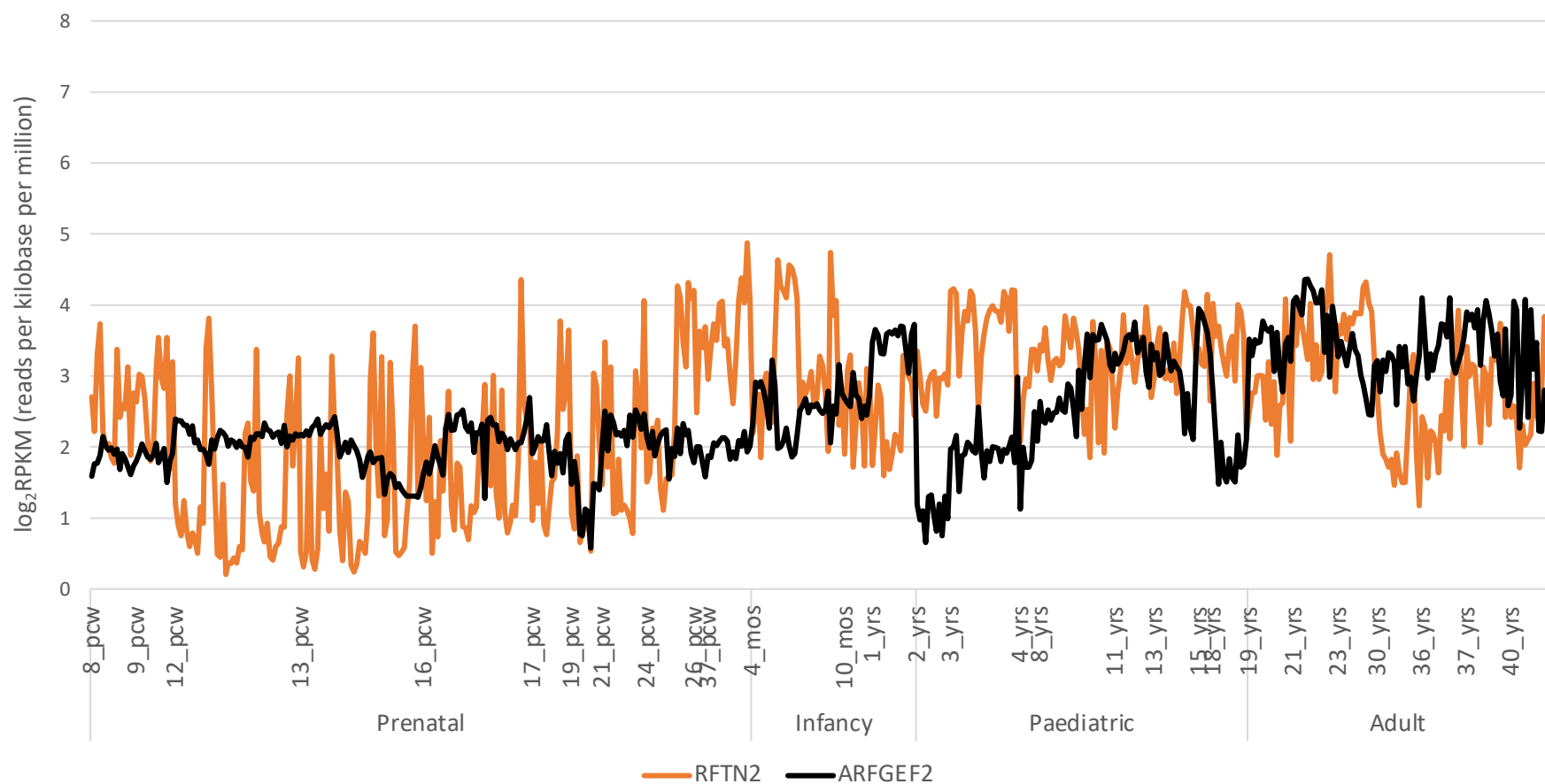

Supplement: Supplementary file 2 — Figure S2: Gene expression of RD familial candidates ROBO1 and CEP63, GWAS candidates RFTN2 and ARFGEF2 in glial cell types (a) and across different stages of human brain development (b). Astro, astrocyte; Micro, microglia; Oligo, oligodendrocytes; OPC, oligodendrocyte precursor cells; Endo, endothelial cells; RB, red blood lineage cells; PC, pericyte; SMC, smooth muscle cells; VLMC, vascular leptomeningeal cells; pcw: post‐conception weeks; mos: months; yrs: years; (a): expression data extracted using Cross‐species dot plots (http://resources.sestanlab.org/PFC/) with a minimum expression at the 5% level; (b): expression data extracted from the Atlas of the Development of Human Brain (http://brainspan.org/rnaseq/search/index.html). [file GBB-23-e12899-s003.pdf]
